# An optimized method for the bio-harvesting of microalgae*, Botryococcus braunii,* using *Aspergillus* sp. in large-scale studies

**DOI:** 10.1016/j.mex.2018.07.010

**Published:** 2018-07-23

**Authors:** Khalid A. Al-Hothaly

**Affiliations:** Department of Biotechnology, Faculty of Science, Taif University, Saudi Arabia

**Keywords:** Bio-harvesting of *B. braunii*, Bio-harvesting, Flocculation, Microalgae, *Aspergillus*, Large-scale

## Abstract

The use of fossil fuels which are derived from non-renewable sources has been linked to global warming, adverse human health effects and environmental pollution. Consequently, there is a need to develop alternative sources of fuel that are renewable and more environment-friendly. Biofuel (biodiesel), produced from microalgae such as *Botryococcus braunii* is an alternative energy source, that is renewable (because algae can be cultured as needed), more biodegradable with lower global warming potential compared to fossil fuels. However, the use of microalgae is hampered by high costs associated with the production and harvesting of microalgal biomass in large-scale studies. In this article;

• A robust and cost-effective method was developed for harvesting *B. braunii*
• Optimized *Aspergillus* sp.: *B. braunii* ratio (1:40) was used to bio-flocculate up to 97% of cultured microalgae in both small and large-scale studies (250 L)
• No damage to the harvested microalgal biomass (validated by pyrolysis) was observed with the harvested biomass being suitable for any desired downstream application.

## Graphical abstract

**Figure:**
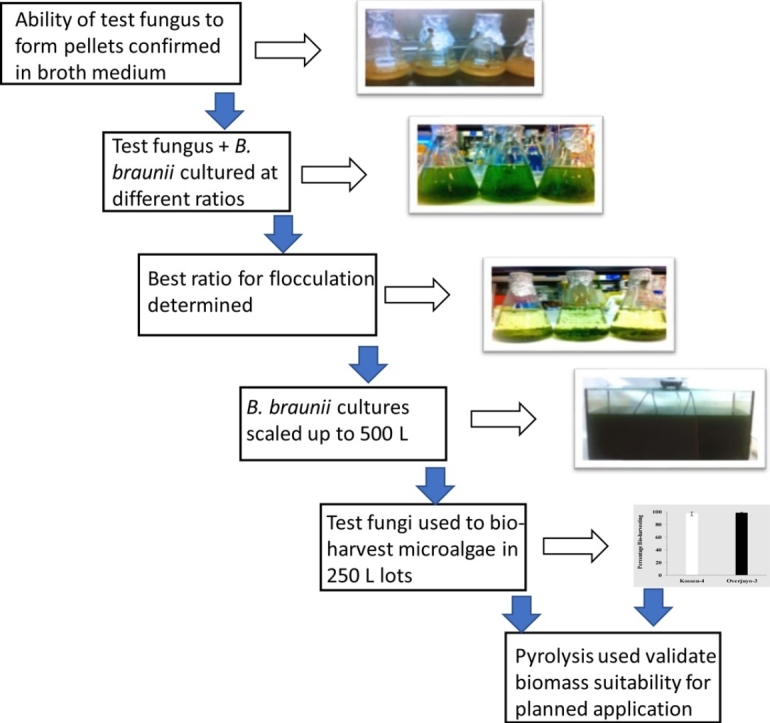

## Specifications table

**Table:** 

Subject area	• Agricultural and Biological Sciences
More specific subject area	*Microalgae*
Method name	*Bio-harvesting of B. braunii*

## Method details

### Background

Global warming caused by the utilization of fossil fuel resources for domestic and industrial purposes is a great challenge in the modern world from an environmental perspective. Combustion of fossil fuels generates CO_2_, which has been implicated in global warming. The extensive use of fossil fuels can result in their accidental release into the environment causing pollution. Fossil fuel pollutants are toxic, carcinogenic and recalcitrant and present significant risks to public health [1]. They are also finite resources and not a sustainable energy source (non-renewable). Some microalgae such *Botryococcus braunii* can be a viable alternative source of fuel, such as biodiesel and bio-oil, which are highly degradable [2]. This is because they are non-food feedstocks and are therefore not affected by co-demand by human population for use as food. Their ability to convert CO_2_ to carbon-rich compounds (including biodiesels) is also greater than that of traditional oleaginous crops [3,4]. They are renewable sources of fuel because they can be easily cultured within a comparably short time-frame unlike fossil fuels, which were formed over millions of years.

To fully exploit this potential, efficient and economical methods of culturing and harvesting cultivated microalgae must be developed. There have been multiple studies designed to determine the best growth medium for culturing specific groups of microalgae. These include the use of Conway and f/2 media for the culture of marine microalgae such *Chlorella* and *Dunaliella* sp. [5] and wastewater for species such as *Chlorella* and *Scenedesmus* [6,7]. Harvesting of microalgae can account for up to 50% of the total cost of biofuel production due to its energy-intensive nature [8]. Different methods of harvesting microalgae such as thermal drying, spray drying, centrifugation and filtration are known. However, these methods are either time consuming, require large drying areas, can damage the microalgae, energy-intensive or costly [[9], [10], [11]].

One innovative and cost-effective method of harvesting microalgae is by bio-flocculation. Some filamentous fungi can form pellets when grown in solution alone or with microalgae naturally without any chemical inducement. These pellets will sink to the bottom of the growth medium and are therefore easily harvested. When introduced into a micro-algae culture, these fungal species will trap the microalgae, forming pellets which settle at the bottom of the growth tank. These have been successfully carried out in a number of studies on some microalgae but at small scale levels [12] and may require optimization of C/N ratios for optimal growth, as practised in aquaculture bio-flocculation [13]. Oil (lipids) can then be extracted from the harvested biomass using different methods such as chloroform-methanol, hexane and supercritical CO_2_ lipid extraction methods [14,15]. A great challenge for most scientific studies is a successful translation of small-scale studies into large-scale studies. In this study, a protocol that was successfully developed and optimized to bio-flocculate a bio-diesel producing micro-algae, *B. braunii,* from a small 1 L scale to large 250 L scale experiments is described. Data that showed that bio-flocculation did not have any significant effect on the microalgae characteristics, with the harvested micro-algae being suitable for further downstream applications are presented.

## Selection of fungal species

The fungal isolates selected for use in harvesting *B. braunii* must be able to grow in the form of pellets (ball-like forms). It was, therefore, necessary to test any prospective fungal candidate for this ability. In this study, the ability of the strains of *Aspergillus fumigatus* (obtained from RMIT’s local repository of cultures) to form pellets was tested using the simple procedures described in the sections (a) and (b).

## Culturing of fungal species

**Fig. 1:**
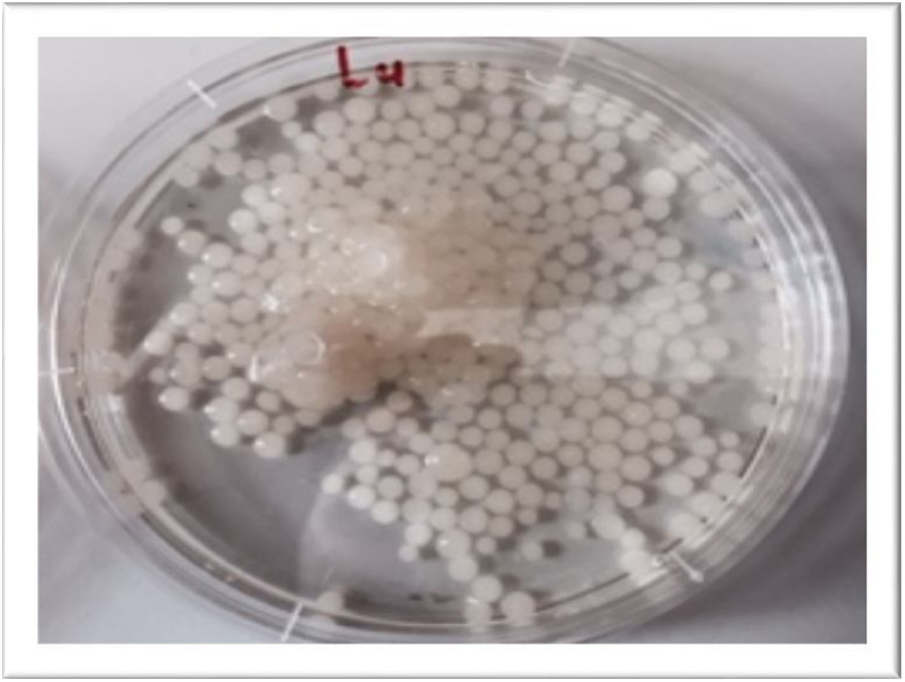
Pellet formation by *Aspergillus* sp.
  (b) **Pelletization in algal broth;**
    It was important to ensure that the selected fungal candidate(s) could also grow and form pellets in the medium used for culturing *B. braunii* (or any other chosen microalgae). This was carried out following the protocol described in this section.
    (i) *B. braunii* was cultured in BG11 media for up to 4 days at 25 °C and 100 rpm by inoculating 500 ml of BG11 medium with 0.04 g L^−1^ of microalgae culture.
    (ii) BG11 medium was prepared by adding different compounds into 1 L flask. The compounds and concentrations (in g L^−1^) used were; NaNO_3_, 1.5, MgSO_4_.7H2O, 0.075, CaCl_2_.2H_2_0, 0.036, K_2_HPO_4_.3H_2_0, 0.040, C_6_H_8_FeNO_7_, 0.006, C_6_H_8_O_7_, 0.006 and EDTA-NA_2_, 0.006.
      Trace elements were also added as components of the medium. The compounds and concentrations (in g L^−1^) used were; H_3_BO_3_, 0.00286, MnSO_4_.H_2_O, 0.00181, ZnSO_4_.7H_2_O, 0.000222, Na_2_MoO_4_.2H_2_O, 0.00039, CuSO_4_.5H_2_O, 0.000079, Co(NO_3_)_2_.6H_2_O, 0.00049.
    (iii) Inoculated flasks (culture and BG11 medium) were placed on a shaker and illuminated with a cool fluorescence lamp at 54 μmol m^−2^ s^−1^ and incubated for up to 4 days at 25 °C and 100 rpm.
    (iv) Forty (40) millilitres of *Aspergillus* broth culture was added to 360 ml aliquots of *B. braunii* broth using sterile 5 ml tips and pipette. The *B. braunii*-*Aspergillus* broths were incubated on a shaker for 12 h at 25 °C and 100 rpm.
    (v) Fungal: algal broths were assessed after 12 h for (a) the formation of ball-like pellets and (b) the complete disappearance of or substantially reduced green algal colouration.
    (vi) A positive observation of these two features indicated that test fungus could grow and form pellet with the microalgae.
  (c) **Optimization of fungal: algal ratio**
    Prior to carrying out large-scale harvesting of *B. braunii* (or any microalgae of choice), it was important to determine the optimal fungal: microalgae ratio in multiple small-scale studies. In the study with *B. braunii*, this was determined following the protocol described in this section with two strains.
    (i) *B. braunii* cultures were prepared as earlier described in b (i) to b (iii).
    (ii) Up to 1 L of fungal culture was prepared by inoculating PDB with fungal plugs (5 plugs in 100 ml of PDB or fungal spores (100–200 μl in 100 ml of PDB).
    (iii) Fungal: microalgal ratios to be tested were determined. In this study with *B. braunii*, the following fungal: microalgal ratios were used
      (1) :1 (50: 50 ml)
      (2) :10 (20:200 ml)
      (3) :20 (20:400 ml)
      (4) :30 (20:600 ml)
      (5) :40 (20:800 ml)
      (6) :50 (20:1000 ml)
    (iv) The appropriate volume of fungal culture was added to the required volume of microalgae culture.
    (v) Control samples composed of microalgae broth alone without the addition of any fungal culture were prepared.
    (vi) The experiments were set up in conical flasks of appropriate volume or capacity. For example, ratios (1) and (2) were set up in 500 ml flasks, ratios (3) and (4) were set up in 1000 ml flasks and ratios (5)–(6) were set up in 2000 ml (2 L) flasks.
    (vii) All the flasks (treatment and control samples) were set up in triplicates on a shaker at 25°C and 150 rpm with cool light fluorescence at 54 μmol m^−2^ s^−1^. Illumination could be up to 200 μmol m^−2^ s^−1^ depending on experimental needs and microalgae used.
    (viii) Non-destructive samplings were carried out at time 0 and every 4 or 6 h over 120 h by aseptically taking 1–1.5 ml of broth culture supernatant from each flask into sterile 2 ml tubes.
    (ix) Broth culture supernatant sample (1 ml) was added to a cuvette and the OD of culture supernatant measured at two wavelengths (680 and 750 nm)
    (x) Obtained data was recorded and plotted as graphs of OD over time for each ratio (Fig.2).

(a)
  (a) **Pelletization tests on fungal growth medium:**
    (i) Sterile Potato Dextrose Agar (PDA) and Broth (PDB) media (Oxoid) were prepared by the addition of 39 g of the selected media to 1 L of RO water for each medium. Complete medium dissolution was achieved by slowly bring to the mixture to boil.
    (ii) The prepared media were sterilized by autoclaving at 121 °C for 15 min. Sterile PDA medium was allowed to cool to around 50 °C and tetracycline at 0.015 g L^−1^ added to the medium. After being shaken gently to mix, the medium was poured into sterile plates (20 mL per plate).
    (iii) Sterile plates were inoculated with 50–100 μL of *Aspergillus* sp. (or any fungal candidate of choice) spores (from a 50% glycerol stock). Inocula were spread unto the plates with a sterile glass or plastic spreader.
    (iv) Inoculated plates were incubated at 25–30 °C for up to 7 days to allow for profuse fungal species growth (cover most parts of the plate).
    (v) A cork borer was dipped into 70% ethanol, briefly passed through the flames of a Bunsen burner, allowed to cool for 30–60 s and used to punch a plug on the mycelia covered PDA plate.
    (vi) Up to 5 fungal plugs (1 cm^2^) were aseptically added to 100 mL of PDB in sterile 250 mL flasks.
    (vii) Inoculated flasks were placed onto an orbital shake, set to a temperature of 28 °C and shaken at 150 rpm for up to 5 days
    (viii) The formation of balls of fungal mycelia (Fig. 1) was visually assessed after 5 days.

**Fig. 2:**
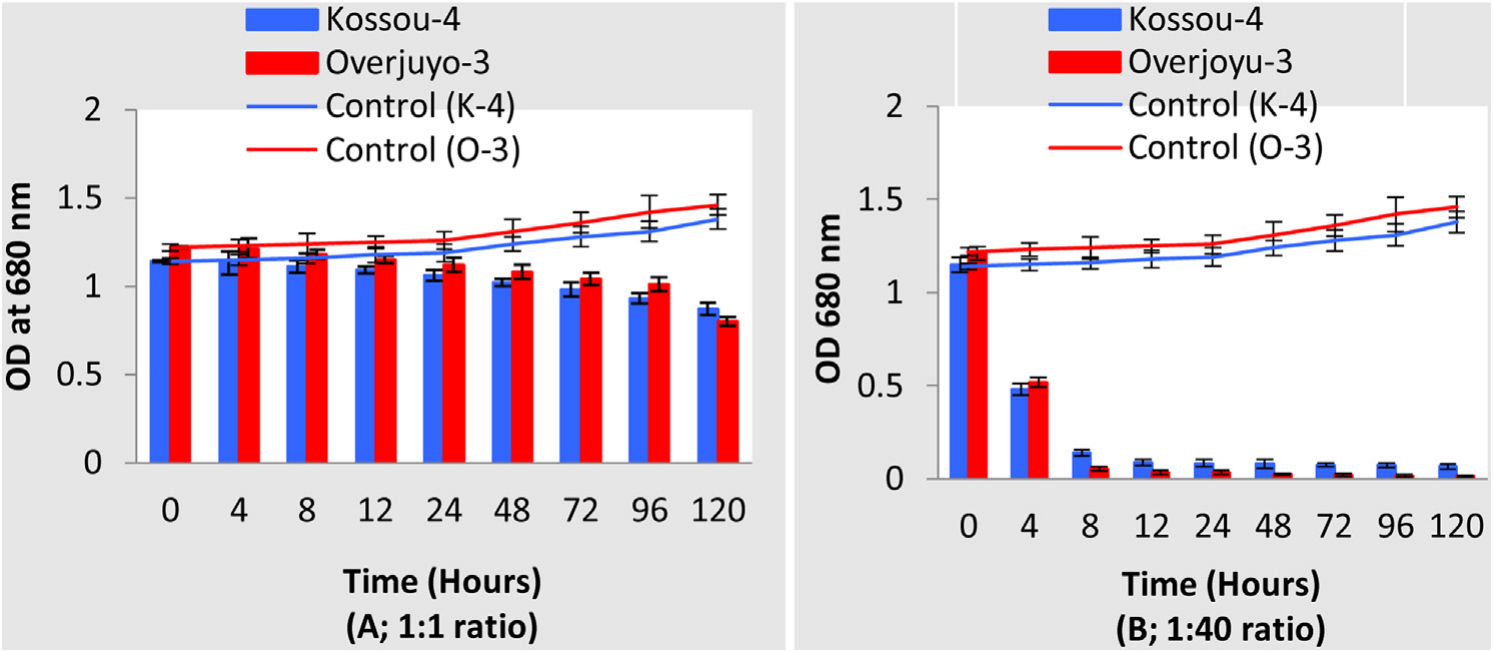
The bio-flocculation of two strains of *B. braunii* (Kossou-4 and Overjuyo-3) in selected ratios (A) 1:1 and B (1:40) of *Aspergillus* sp. to *B. braunii* over 120 h at 680 nm OD in BG11 medium. N = 3.
  (d) **Large-scale bio harvesting**
    The analyses of the result were carried out and the flocculation efficiency (FE) determined with the formula; FE% = [(A–B)/A] ×100. A refers to the OD at time 0, B refers to OD at desired or selected time interval [8]. In the study with *B. braunii* strains, ratio 1:40 was observed to recover about 96–97% of microalgae. Therefore, for the large-scale study, the 1:40 ratio was used. A similar biomass recovery trend was observed at both 680 and 750 nm. Therefore, only the 680 nm OD measurements are presented (Fig. 2). For large-scale bio-harvesting, the protocol described in this section was followed.
    (i) Cultures of *B. braunii* strains (labelled as K1 and O1 and 20 L of each strain) were prepared as earlier described in b (i) to b (iii).
    (ii) Thirty litres of modified BG11 culture broth were prepared for each strain.
    (iii) Two circular fiberglass tanks (600 L capacity each) were cleaned initially with MilliQ grade water or sterile RO water before being flushed twice with sterile water.
    (iv) To each tank was attached multiple air lines to supply filtered air.
    (v) Four hundred and ninety (490) litres of water was added to each tank after which 10 L of modified BG11 culture broth was added.
    (vi) The filtered air was introduced at 19 m^3^ min^−1^ or higher which ensured that aeration caused the medium in the tank to swirl or mix properly.
    (vii) The microalgae in each of the tanks were allowed to grow for 60 days with continuous aeration through the bubbling of air from the bottom and centre of the tank. Cool fluorescence lamps at 54 μmol m^−2^ s^−1^ of light provided continuous illumination. Up to 200 μmol m^−2^ s^−1^ of illumination could be used depending on experimental needs.
    (viii) Fiberglass Tanks were placed in a facility with controlled temperature and ambient temperature was approximately 25 °C.
    (ix) The air lines were regularly checked, and air flow adjusted as required to maintain the flux (mixing) needed for microalgal growth.
    (x) After 60 days of incubation, the 500 L microalgal culture of each strain was divided into two, 250 L volumes.
    (xi) Fungal cultures (*Aspergillus* sp.) were added to each 250 L volume of microalgal culture for each strain to achieve the desired ratio (1:40). The remaining 250 L microalgal culture of each strain was left untreated and designated as CTL.
    (xii) For the 250 L volumes with fungal culture added, growth conditions were maintained by continuous bubbling of air and illumination at 54 μmol m^−2^ s^−1^ of light for 12 h (Fig. 3).

**Fig. 3:**
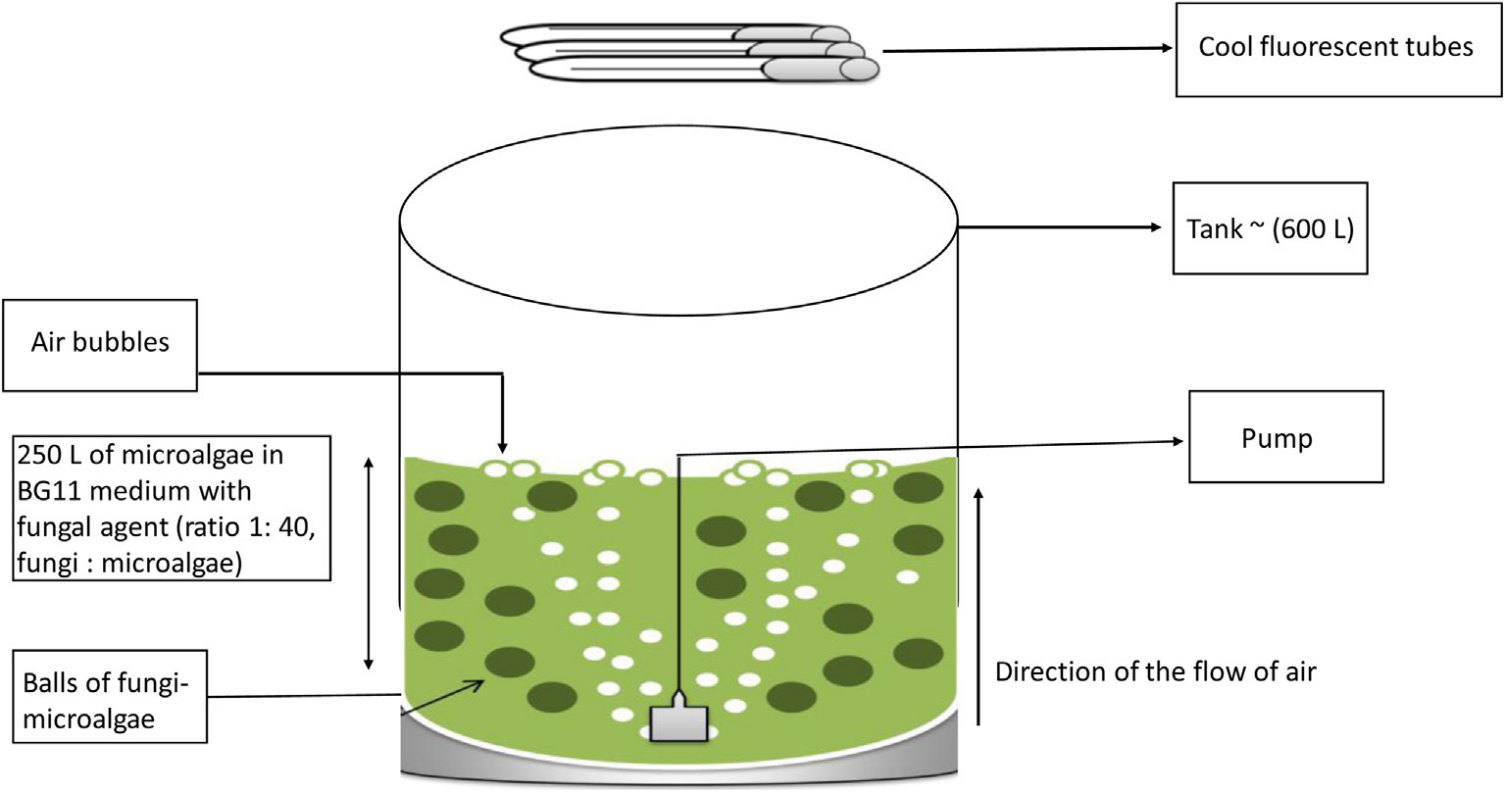
Schematic representation of large-scale (250 L) culture of microalgae, *B. braunii* harvested with *Aspergillus* sp.
    (xiii) After 12 h most of the microalgae had been pelleted with the fungus.
    (xiv) The pellets were harvested by draining the liquid using sieves of different mesh sizes (mesh-size determined by the size of the pellets formed) to trap the pellets.
    (xv) The remaining 250 L volumes (left untreated and designated CTL) were centrifuged and used for validation purposes in pyrolysis.
  (e) **Method validation**
    As the harvested *B. braunii* was likely to be used for biofuel production, it was crucial to show that harvesting these strains with the fungus did not cause any significant alteration in microalgae composition. Bioenergy analyses of the microalgae biomass harvested by flocculation with fungus and that of the microalgae harvested by centrifugation were carried out using pyrolysis (analytical Py–GC–MS and preparative pyrolysis) and compared [2]. Fig. 4 showed that the bioenergy of the biomass harvested with Aspergillus sp. was not significantly different from that of the biomass harvested by centrifugation (P ≥ 0.05). Therefore, the method use for bio-harvesting was highly efficient and would have no adverse impact on the downstream use of the microalgae.

**Fig. 4:**
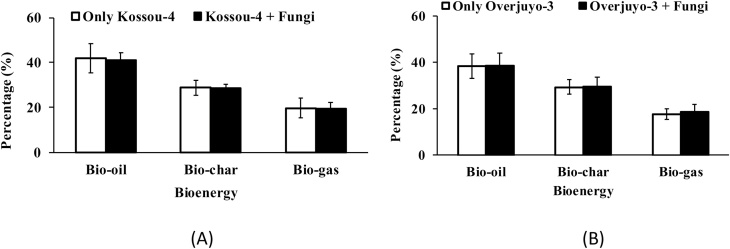
Bioenergy analysis of microalgal biomass harvested with and without fungi (centrifugation).

## References

[1] Kim K.-H., Jahan S.A., Kabir E., Brown R.J. (2013). A review of airborne polycyclic aromatic hydrocarbons (PAHs) and their human health effects. Environ. Int..

[2] Al-Hothaly K.A., Adetutu E.M., Taha M., Fabbri D., Lorenzetti C., Conti R., May B.H., Shar S.S., Bayoumi R.A., Ball A.S. (2015). Bio-harvesting and pyrolysis of the microalgae Botryococcus braunii. Bioresour. Technol..

[3] Wijffels R.H., Barbosa M.J. (2010). An outlook on microalgal biofuels. Science.

[4] Mata T.M., Martins A.A., Caetano N.S. (2010). Microalgae for biodiesel production and other applications: a review. Renew. Sustain. Energy Rev..

[5] Lananan F., Jusoh A., Lam S.S., Endut A. (2013). Effect of conway medium and f/2 medium on the growth of six genera of South China sea marine microalgae. Bioresour. Technol..

[6] Pittman J.K., Dean A.P., Osundeko O. (2011). The potential of sustainable algal biofuel production using wastewater resources. Bioresour. Technol..

[7] Nasir N.M., Bakar N.S.A., Lananan F., Hamid S.H.A., Lam S.S., Jusoh A. (2015). Treatment of African catfish, Clarias gariepinus wastewater utilizing phytoremediation of microalgae, Chlorella sp. with Aspergillus niger bio-harvesting. Bioresour. Technol..

[8] Wrede D., Taha M., Miranda A.F., Kadali K., Stevenson T., Ball A.S., Mouradov A. (2014). Co-cultivation of fungal and microalgal cells as an efficient system for harvesting microalgal cells, lipid production and wastewater treatment. PloS One.

[9] Brennana Liam, Powende Phili (2010). Biofuels from microalgae—a review of technologies for production, processing, and extractions of biofuels and co-products. Renew. Sustain. Energy Rev..

[10] Widjaja A., Chien C.C., Ju Y.H. (2009). Study of increasing lipid production from fresh water microalgae Chlorella vulgaris. J. Taiwan Inst. Chem. Eng..

[11] Leite G.B., Abdelaziz A.E., Hallenbeck P.C. (2013). Algal biofuels: challenges and opportunities. Bioresour. Technol..

[12] Lananan F., Yunos F.H.M., Nasir N.M., Bakar N.S.A., Lam S.S., Jusoh A. (2016). Optimization of biomass harvesting of microalgae, Chlorella sp. utilizing auto-flocculating microalgae, Ankistrodesmus sp. as bio-flocculant. Int. Biodeterior. Biodegrad..

[13] Bakar N.S.A., Nasir N.M., Lananan F., Hamid S.H.A., Lam S.S., Jusoh A. (2015). Optimization of C/N ratios for nutrient removal in aquaculture system culturing African catfish, (Clarias gariepinus) utilizing bioflocs technology. Int. Biodeterior. Biodegrad..

[14] Halim R., Gladman B., Danquah M.K., Webley P.A. (2011). Oil extraction from microalgae for biodiesel production. Bioresour. Technol..

[15] Lee J.-Y., Yoo C., Jun S.-Y., Ahn C.-Y., Oh H.-M. (2010). Comparison of several methods for effective lipid extraction from microalgae. Bioresour. Technol..

